# Attitudes toward pornography and gender differences among Spanish young adults

**DOI:** 10.3389/fpsyg.2026.1719257

**Published:** 2026-02-17

**Authors:** Mariela Velikova, M. Carmen Terol-Cantero, Maite Martín-Aragón, Carolina Vázquez-Rodríguez

**Affiliations:** Department of Behavioral and Health Sciences, Miguel Hernández University of Elche, Elche, Spain

**Keywords:** cluster analysis, gender dynamics, pornography consumption, sexual attitudes, transgressive content, youth

## Abstract

Pornography consumption is a widespread practice among young people that influences both attitudes and sexual behavior, and its analysis is essential to understanding the factors that shape gender dynamics and sexual socialization today. The present study aimed to examine attitudes toward pornography, frequency of consumption, and preferred content in a sample of Spanish youth aged 18 to 29 years. Two studies were conducted: the first (*N* = 285) assessed the psychometric properties of the Pornography Attitudes Scale confirming its validity and reliability. The second (*N* = 1,000) evaluated attitudes toward pornography (fun, arousal, curiosity, and social acceptance) and the intention to seek specific types of content (explicit, BDSM, violence, and fetishism). Analyses included descriptive techniques, confirmatory factor analysis, non-parametric testing, and cluster analysis. Results showed that men reported higher consumption and more favorable attitudes, whereas women displayed greater critical sensitivity. Cluster analysis revealed three distinct profiles (conventional consumers, transgressive consumers, and non-consumers/critics), highlighting the heterogeneity of youth consumption. Overall, findings confirm the validity of the EAP and underscore the existence of a subgroup with a marked interest in transgressive content, with clear implications for gender-related risks.

## Conceptualization of mainstream pornography

Pornography has been defined in diverse ways within the literature. In general terms, it is described as digitally distributed audiovisual material that is easily accessible, sexually explicit, and primarily intended to provoke arousal or pleasure (Ballester et al., 2022). Mainstream pornography is further distinguished as the dominant form of consumption, characterized by free and easy access and unlimited availability (Canet Benavent and Martínez Martínez, 2023). More recent definitions incorporate the notion of consent in its production (Ashton et al., 2019), yet critical perspectives emphasize that such views often obscure its patriarchal orientation, the centrality of women as performers for male consumption, and the recurrent presence of sexual violence and dynamics of domination (Cobo, 2020; Díaz Hernández et al., 2024; Torrado Martín-Palomino et al., 2021).

Although pornography constitutes a heterogeneous phenomenon encompassing a wide range of contents, formats, and user experiences, empirical research has consistently defined it as sexually explicit audiovisual material intended to provoke sexual arousal or pleasure, while also documenting substantial variability in production contexts and represented practices (McKee et al., 2020; Jahnen et al., 2022; Merlyn et al., 2020; Willoughby and Dover, 2022).

While pornography is widely recognized as a social phenomenon, the scope of its content and the implications of its widespread availability are often overlooked. Pornography brings together elements of desire, domination, and violence (Cobo, 2019). Mainstream productions tend to highlight gendered power inequalities, reinforcing the subordination of female pleasure to male pleasure and frequently eroticizing violence (Cerbara et al., 2023). Contemporary pornography is disseminated across multiple platforms and has been shown, in several content analysis studies, to include a high prevalence of violent or degrading practices in certain widely consumed mainstream materials, distributing images and videos in which explicit sexual violence is predominantly directed at women through practices that denigrate and objectify them (Ballester et al., 2023; Merlyn et al., 2020; Torrado Martín-Palomino et al., 2024). Content analyses of popular mainstream pornographic videos have reported that more than 60% of depicted sexual practices involve acts of violence against women, where their resistance is overridden, their pain is eroticized, and they are portrayed as being forced into sexual acts (Ballester et al., 2022). For the purposes of the present study, the theoretical framework focuses specifically on heterosexual mainstream pornography, which constitutes the dominant form of consumption examined in most of the content analyses cited, and does not seek to generalize its conclusions to LGBTQ+ pornographic content.

This type of content has been shown to prioritize performance-oriented and genital-focused representations of sexuality, often marginalizing affective, relational, and contextual dimensions in favor of normative sexual scripts (Rodríguez et al., 2018; Alario, 2018; Ballester et al., 2022). At the same time, it is important to note that for many young people pornography is primarily consumed as a form of entertainment or leisure, particularly in contexts where formal affective-sexual education is limited, an issue that is addressed in greater depth in the following section.

Such content also conveys ideals of masculinity and femininity, body image, sexual performance, and notions of desirability and acceptability. The consumption of pornography is therefore closely linked to the development of the affective-sexual sphere of young people and adolescents. In the absence of comprehensive affective-sexual education, pornography often becomes an informal source of information, disseminating explicit material about types of sexual relations, scenarios and scripts of sexual acts, potential partners, and positions (García and Pollán, 2024; Torrado Martín-Palomino et al., 2024; Torres et al., 2023). As a result, mainstream online pornography, as the most widely consumed form, plays a significant role in shaping ideas about gender and sexuality.

Pornography also functions as an informal educator, teaching many children and young people what is perceived as “normal” in sexual acts and what position women should occupy within them. Such content, viewed by millions of minors, contributes to shaping sexual scripts, addiction and everyday understandings of intimacy, often through exposure to repeated visual scenes (Alario, 2018; Jahnen et al., 2022).

In this sense, pornography operates as a key agent of socialization for adolescents and young people, shaping “men” who internalize norms of dominant masculinity and “women” who are positioned as submissive sexual objects, thereby devedoping and reinforcing a hegemonic masculinity grounded in inequality (Alario, 2018; de Heer et al., 2021; Delicado-Moratalla and Perez, 2024).

The sexualization and objectification of women reach one of their most extreme expressions in the link between pornography and prostitution, particularly insofar as prostitution is eroticized through countless pornographic videos with millions of views—predominantly consumed by men—on mainstream platforms such as PornHub (Alario, 2018; Torrado Martín-Palomino et al., 2024). Some authors have argued that pornography can be understood as a form of consuming prostitution at low cost, insofar as it normalizes sexual scripts that can later be enacted in prostitution contexts, with psychological and social consequences primarily affecting prostituted women (Alario, 2018; Farley, 2017).

A growing body of research has documented a consistent association between the frequency of pornography consumption and the use of prostitution, particularly among young men, as well as the use of prostitution as a means of enacting sexual fantasies learned through pornography (Ballester and Orte, 2019; Gomes et al., 2018; Ranea Triviño, 2016, 2019; Instituto de la Juventud (INJUVE), 2021; Ariño, 2022; García and Cuervo Pollán, 2023). In this context, the notion of “transfer” refers to the reproduction, within prostitution settings, of sexual scripts based on domination, objectification, and female sexual availability that are widely depicted in mainstream pornographic content.

Empirical evidence indicates that these dynamics are associated with a high prevalence of physical and sexual violence, as well as severe psychological sequelae—including post-traumatic stress disorder, dissociation, depression, and suicidal behaviors—among prostituted women (Farley, 2017). From the perspective of male consumers, frequent pornography use has been associated with greater objectification of women and more tolerant attitudes toward violence against them, contributing to the normalization of such practices (Hald et al., 2010; Wright et al., 2016; Mestre-Bach et al., 2024).

Within this framework, we argue that the eroticization of prostitution through pornography cannot be understood solely as an extension of “sex work,” but rather as part of a broader process of sexual socialization that reinforces gender inequalities and power dynamics previously described in the manuscript.

## Pornography consumption attitudes and consequences

There is an increasing trend in the consumption and social acceptance of pornography, an issue that has generated open debate in contemporary society (Ballester et al., 2022; FAD Juventud, 2023; Martínez-Fernández et al., 2025; Save the Children España, 2020). Pornography is consumed by diverse groups, including men and women, adults and minors, with evidence indicating patterns of use beginning before the age of 10 and becoming normalized around the age of 13, with the majority of consumers between 12 and 18 years old (Colom et al., 2024; García and Pollán, 2024; Martínez et al., 2023; Merlyn et al., 2020; Torrado Martín-Palomino et al., 2024). According to data from PornHub^1^ Spain ranks fourth in Europe and twelfth worldwide in pornography consumption. A recent study comparing Spanish and Mexican adolescents reported higher levels of pornography use among Spanish participants, together with a higher prevalence of behaviors considered sexually risky, such as non-condom use and sexual activity after alcohol or drug use (Villena-Moya et al., 2024). In line with these findings, other studies have documented associations between pornography consumption and engagement in unwanted sexual practices, as well as differences in condom use and sexual decision-making among young people (Díaz Hernández et al., 2024; Jahnen et al., 2022).

Although studies indicate that pornography is consumed by both sexes, it is predominantly consumed by males, with prevalence estimates up to 98.3% depending on the source (Ballester et al., 2022; Díaz Hernández et al., 2023; Durante, 2022; FAD Juventud, 2023; Gutiérrez García, 2024; Sanz-Barbero et al., 2023; Torrado Martín-Palomino et al., 2024). Research further shows that individuals who consume pornography more frequently tend to hold more accepting and positive attitudes toward pornographic content (Noll et al., 2022; Rasmussen et al., 2018; Willoughby et al., 2016). Across genders, men consistently report greater acceptance of pornography (Maas et al., 2018; Mattebo et al., 2014; Negy et al., 2018; Noll et al., 2022).

Several studies also highlight that intensive and frequent use of pornography, particularly among men, is associated with a higher risk of problematic consumption and cybersex addiction (Ballester and Orte, 2019; Biota et al., 2022; García et al., 2018; Gomes et al., 2018; Koletic et al., 2021). In this context, “problematic consumption” refers to patterns of pornography use perceived by individuals themselves as difficult to control or potentially addictive, which may interfere with their well-being or interpersonal relationships, rather than to clinically diagnosed addiction (Ballester et al., 2022). This pattern appears more pronounced among young men compared to young women, underscoring gender as a key factor in pornography consumption (Farré et al., 2020; López-Pinar et al., 2025; Noll et al., 2022).

A growing body of research has examined the correlates and consequences of pornography consumption, particularly in shaping sexual attitudes and behaviors. When comparing pornography consumers with non-consumers, evidence shows that non-consumers are more likely to reject behaviors such as spanking, hair pulling, or the use of obscene language during sexual activity (Merlyn et al., 2020). Other studies have linked pornography use with sexual violence and rape myth acceptance (Hald et al., 2010; Noll et al., 2022), adherence to traditional gender roles (Koletić, 2017), and the objectification of women as sexual objects (Mestre-Bach et al., 2024; Vandenbosch and van Oosten, 2017). The frequent pornography use is reliably associated with sexually aggressive behavior, particularly in the case of violent pornography (Léon et al., 2025; Malamuth et al., 2000). Additional findings suggest associations with reduced sexual arousal, greater sexual uncertainty, unsafe sexual practices, higher numbers of sexual partners, negative self-image, risky sexual attitudes, poor mental health outcomes, substance use, aggressive sexual styles, and violence (Braithwaite et al., 2015; García and Pollán, 2024; Koletic et al., 2021; Landripet et al., 2025; Merlyn et al., 2020; Peter and Valkenburg, 2016; Willoughby et al., 2016).

At the same time, a systematic review by Komlenac and Birke (2025) indicates that pornography use can also be associated with better sexual functioning, including greater desire, arousal, and orgasm frequency, while problematic or compulsive use tends to correlate with sexual dysfunctions. In line with this, other studies report both negative and positive consequences of pornography consumption, ranging from problematic use to enhanced sexual well-being (Koletic et al., 2021; Komlenac and Birke, 2025; Landripet et al., 2025; Merlyn et al., 2020).

Although mainstream pornography has been widely criticized for its gendered power dynamics, the literature also highlights that pornography consumption is a heterogeneous phenomenon, and that for many young people it is experienced as a recreational practice or a source of sexual curiosity, with ambivalent effects that are discussed in later sections. Research focusing on favorable attitudes toward pornography often emphasizes its role in sexual well-being. Between 75 and 90% of respondents describe pornography use as recreational, curiosity-driven, and enjoyable, highlighting potential benefits such as enhanced sexual knowledge, skills, enjoyment, arousal, interest in sex, and broader social acceptance (Bothe et al., 2021; Koletic et al., 2021; LeBlanc and Trotter, 2022; Monferrer and Flor, 2015). Similarly, some studies suggest that young people may view pornography as leisure, a way to learn specific techniques, and a means of exploring identity and orientation (McCormack and Wignall, 2016). Nevertheless, other research highlights that young people are aware of its limitations as a source of information, pointing to the need for improvements in sex and relationships education (Litsou et al., 2024).

Although both men and women report negative and positive experiences, evidence suggests that men are more likely to progress from favorable attitudes toward problematic consumption, addiction, or abuse of pornography (Ballester and Orte, 2019; Biota et al., 2022; Farré et al., 2020; García et al., 2018; Gomes et al., 2018; Koletic et al., 2021; López-Pinar et al., 2025; Noll et al., 2022). For these reasons, the phenomenon of pornography—fully normalized and widely accessible—has become a topic of particular social relevance, leading to the development of instruments to assess patterns of consumption, its consequences, and people’s attitudes toward it.

## Pornography assessment: attitudes, use, and abuse

In the field of pornography assessment, some studies have examined population attitudes, although most have focused on instruments measuring frequency of use, consumption patterns, or indicators of abuse. To identify tools specifically assessing attitudes, we conducted a targeted literature review in Scopus (2010–2022; English/Spanish) using a combination of terms related to pornography, scales, and psychometric evidence. The search was complemented with backward citation chasing, contact with original authors, and manual searches in a specialized journal. Only self-report instruments explicitly addressing pornography and providing psychometric evidence (e.g., internal consistency and at least one validity indicator) were included, while theoretical, intervention, clinical/forensic, or relationship-focused studies were excluded. The initial search yielded 135 records, with 16 additional studies identified through complementary sources. After screening and applying the eligibility criteria, 28 studies were retained. Given the targeted nature of the review, data were synthesized narratively and organized in a comparative table including information on construct, population, items/subscales, factor structure, reliability, validity evidence, language, and country.

Of the 28 studies reviewed, three focused on the assessment of attitudes toward pornography, two on pornography use, and the remainder addressed abuse or problematic consumption. This initial classification was based on the stated aim of each instrument and the type of items included. Attitudinal measures, for example, contained impersonal items assessing general opinions, such as “Pornography is fun or boring” (Monferrer and Flor, 2015) or “Pornography on the Internet improves people’s sex life” (Noll et al., 2022). Items such as “How often do you see sexually explicit material/pornography?” (Szymanski and Stewart-Richardson, 2014) referred specifically to frequency of use, while others such as “I use it to expand my knowledge about sex” (Leon-Larios et al., 2019) captured personal motivations for consumption. These were classified as use or consumption measures. Finally, items addressing problematic patterns included statements such as “How often do you find it difficult to stop accessing these websites when you are online?” (Downing et al., 2014) or “I felt I had to watch more and more porn to get satisfaction” (Bothe et al., 2017), which were categorized as indicators of problematic or abusive consumption.

Within the review, seven studies presented instruments adapted for Spanish-speaking populations (Leon-Larios et al., 2019; Merlyn et al., 2020; Monferrer and Flor, 2015; Serpa-Barrientos et al., 2023; Tarragón et al., 2024; Valenzuela Romero et al., 2024; Villena-Moya et al., 2024). Of these, four were administered in Spain. However, only the study by Monferrer and Flor, 2015 specifically addressed attitudes or opinions toward pornography, while the others focused on personal use and problematic consumption.

## The purpose of this work

Theoretical and empirical research has identified several factors influencing the demand for pornography and its widespread prevalence. Technological advances have substantially increased its accessibility and frequency of use, accompanied by varying levels of acceptance and diverse consequences for the population. In response, much of the research in this field has sought to develop reliable and valid instruments capable of capturing the scope of this phenomenon, assessing not only population attitudes but also the consequences and frequency of pornography consumption.

The present work aims to examine attitudes toward pornography consumption in a Spanish sample using the Attitudes Toward Pornography Scale (EAP) (Monferrer and Flor, 2015). The first study will confirm the psychometric properties of this scale, thereby providing a practical tool for assessing attitudinal beliefs about pornography. The second study will apply the validated instrument to evaluate Spanish participants’ attitudes toward pornography, its frequency of consumption, and preferred content. Based on previous research, it is hypothesized that men will report higher consumption and more favorable attitudes toward pornography than women. Furthermore, men are expected to show greater overall acceptance of pornography and increased tolerance toward violent pornographic content. Additionally, differentiated profiles of pornography consumption and attitudes will be explored among young people.

In the following sections, each study is presented separately, with details on its sample, administered questionnaires, type of analysis, and results. Before that, we provide an overview of the common sociodemographic information collected and the procedures applied in both studies. Data were collected online between October 2023 and May 2024. In both studies, participants reported their sex, sexual orientation, age, nationality, institution or group affiliation, marital status, educational level, current situation, socioeconomic class, religious beliefs, and political views. All personal data were anonymized and stored on secure servers to ensure confidentiality. Prior to completing the questionnaires, participants were required to provide electronic informed consent after receiving a detailed explanation of the study’s objectives, purpose, and relevance. Both studies have been conducted within the framework of a research project funded by the Department of Education, University, and Employment of the Generalitat Valenciana (CIACO/161/2022), and received a favorable ethical approval report from the Office for Research (O. I. R., Research Office Register: 191211112032; Reference: DCC. MMG.03.19) at Miguel Hernández University. Statistical analyses were performed using Jamovi software (Version 2.2) and the R environment (Version 4.0.1), with the *Lavaan* package.

## First study

The sample consisted of 285 young people aged 18 to 29 (*M* = 20.9; SD = 2.56), selected through purposive non-probabilistic sampling, who completed the survey using a Google Forms questionnaire. Of the participants, 49.8% (*n* = 142) were women and 50.2% were men (*n* = 143). In terms of education and current situation, 29.4% (*n* = 84) had completed secondary or high school, and 69.8% (*n* = 199) held university-level education; 84.6% were students and 15.4% were employed professionals. Additionally, 65% (*n* = 185) identified as belonging to a lower or lower-middle social class. Participants were recruited using a convenience sampling method and the snowball technique.

The Attitudes toward Pornography Scale (EAP) (Monferrer and Flor, 2015) was administered. The original scale consists of 32 items distributed across four subscales: Fun (items 1, 2, 3, 6, 10, 11, 12, 15, and 20), Arousal (items 4, 5, 13, 14, 18, 22, and 23), Curiosity (items 9, 16, 17, 19, 31, and 32), and Acceptance (items 7, 8, 21, 24, 25, 26, 27, 28, 29, and 30). “Please indicate the option that best reflects your degree of agreement or disagreement with each of the following statements about pornography.” Responses were recorded on a 5-point Likert scale (1 = Strongly Disagree, 2 = Disagree, 3 = Neither Agree nor Disagree, 4 = Agree, 5 = Strongly Agree). Higher scores reflect a more favorable attitude toward pornography. In the original study, the Cronbach’s alpha for the total scale was 0.943, and the subscales showed values ≥ 0.759 (range: 0.759–0.885).

## Data analysis

The statistical analyses were performed in R (v4.3.2). A confirmatory factor analysis (CFA) was conducted with the *lavaan* package using the WLSMV estimator, suitable for ordinal and non-normally distributed data (Li, 2016). Preliminary item means and standard deviations were calculated, and reliability indices obtained with *semTools*.

Model fit was assessed according to international standards (Brown, 2015; Hooper et al., 2008; Kline, 2010) using multiple criteria: CFI and TLI ≥ 0.90 (≥ 0.95 excellent), RMSEA ≤ 0.08 (McDonald and Ho, 2002) or ≤ 0.07 (Steiger, 2007) with narrow CI and p-close ≥ 0.05, and SRMR ≤ 0.08. Incremental indices (RNI, NFI, IFI, NNFI) were also examined, with values > 0.90 indicating good fit (McDonald and Ho, 2002). Parsimony was evaluated through PNFI, with ≥ 0.50 acceptable and ≥ 0.90 excellent (Mulaik et al., 1989). As WLSMV tends to underestimate RMSEA (Xia and Yang, 2019), greater emphasis was placed on SRMR, which is robust across estimators (Shi and Maydeu-Olivares, 2020).

Internal consistency was assessed using Cronbach’s *α* and McDonald’s *ω*, with ≥ 0.70 considered acceptable (Nunnally and Bernstein, 1994). Convergent validity was tested with the average variance extracted (AVE), with values > 0.50 deemed adequate (Fornell and Larcker, 1981).

## Results

The four-factor model demonstrated a satisfactory fit to the data (χ^2^(458) = 1067.114, *p* < 0.001). The fit indices indicated very good global fit, with CFI = 0.988, TLI = 0.987, SRMR = 0.068 within acceptable limits, and RMSEA = 0.069 (90% CI [0.064–0.075]) falling in the adequate range. In addition, incremental indices also reflected an optimal model specification (RNI = 0.988, NFI = 0.980, IFI = 0.988, NNFI = 0.987). Finally, the parsimony index (PNFI = 0.900) suggested that the model achieved this level of fit with a simple and efficient structure, reinforcing its theoretical adequacy. Overall, these results provide robust support for the structural validity of the proposed model.

All factor loadings were positive, statistically significant (*p* < 0.001), and reflected a strong association between the items and their respective latent constructs. For the *Fun* factor, standardized loadings (*β*) ranged from *β* = 0.552 (item 12) to *β* = 0.783 (item 20), indicating strong internal consistency. In *Arousal*, loadings ranged from *β* = 0.506 (item 14) to *β* = 0.891 (item 18). For *Curiosity*, values ranged from *β* = 0.525 (item 32) to *β* = 0.841 (item 17), while in *Acceptance* they ranged from *β* = 0.456 (item 25) to *β* = 0.903 (item 7). Most loadings exceeded the 0.60 threshold, and several were above 0.75, indicating excellent convergent validity. Although some items, such as item 25, displayed more moderate loadings, all remained within acceptable ranges, supporting the adequacy of the proposed model (Table 1).

**Table 1:** Factor loadings and reliability of EAP items.

Item	M (SD)	*β*	*R* ^2^	*α*	*ω*
Fun				0.86	0.87
1. It is fun.	2.43 (1.09)	0.758	0.575		
2. It is not satisfying.*	2.96 (1.09)	0.599	0.359		
3. It is a good distraction in free time.	2.14 (1.11)	0.689	0.475		
6. It is boring.*	2.91 (0.94)	0.763	0.582		
10. It is pleasurable.	3.19 (1.04)	0.775	0.601		
11. I t is not a good way to set aside problems.*	4.33 (0.98)	0.613	0.376		
12. Watching it makes one feel guilty.*	3.15 (1.09)	0.552	0.305		
15. Watching it is a waste of time.*	3.41 (1.01)	0.691	0.478		
20. It makes life more cheerful.	2.17 (1.05)	0.783	0.613		
Arousal				0.83	0.83
4. It is exciting.	3.44 (1.12)	0.793	0.628		
5. It can be included in couple relationships.	2.95 (1.18)	0.559	0.313		
13. It is a good way to stimulate oneself before a sexual relationship.	2.24 (1.07)	0.550	0.302		
14. It removes feelings from sexual relationships.*	3.06 (1.23)	0.506	0.256		
18. It is disgusting.*	3.14 (1.10)	0.891	0.794		
22. It is attractive.	3.18 (1.08)	0.706	0.499		
23. Watching it can be a good way to stimulate oneself when alone.	3.64 (1.06)	0.800	0.639		
Curiosity				0.77	0.79
9. It creates unrealistic expectations.*	4.59 (0.70)	0.816	0.666		
16. It increases interest in learning more about sex.	3.04 (1.16)	0.686	0.471		
17. It helps improve sexual practices.	2.41 (1.20)	0.841	0.708		
19. It teaches nothing new.*	2.99 (1.12)	0.641	0.410		
31. It can make you feel more confident.	2.14 (1.02)	0.537	0.289		
32. It helps reduce prejudices about sex.	2.18 (1.16)	0.525	0.276		
Acceptance				0.90	0.91
7. More people should watch it.	1.81 (0.96)	0.903	0.815		
8. It is a social disgrace.*	3.17 (1.23)	0.768	0.589		
21. It is a degradation of the sexual act.*	3.60 (1.13)	0.827	0.684		
24. It should be illegal.*	2.89 (1.24)	0.833	0.694		
25. It is like being unfaithful to one’s partner.*	2.23 (1.18)	0.456	0.208		
26. People who watch it are perverted.*	2.32 (1.10)	0.665	0.442		
27. Watching it is normal.	3.25 (0.96)	0.724	0.525		
28. It is dangerous.*	4.00 (1.11)	0.747	0.558		
29. It is disrespectful.*	2.89 (1.14)	0.782	0.612		
30. It discriminates against women.*	3.69 (1.23)	0.807	0.651		

M, mean; SD, standard deviation; β, standardized factor loading; *R*^2^, coefficient of determination; α, Cronbach’s alpha; ω, McDonald’s total omega; *, reverse-scored item.

The residual variances of the items (e^2^), which represent the proportion of variance not explained by the latent factor, ranged from 0.185 (item 7) to 0.792 (item 25). Lower values indicate greater explanatory precision of the factor, whereas higher values reflect greater item specificity or measurement error. Overall, the model accounted for between 21% (*R*^2^ = 0.208) and 82% (*R*^2^ = 0.815) of the item variance, further supporting its structural robustness.

The estimated variances of the latent factors were 0.575 for Fun, 0.628 for Arousal, 0.666 for Curiosity, and 0.815 for Acceptance, all statistically significant (*z* > 8.8; *p* < 0.001). Acceptance showed the highest variance, suggesting greater response variability and, therefore, higher sensitivity of the construct to capture individual differences. All factor covariances were significant (*p* < 0.001), with standardized correlations ranging from 0.811 to 0.958. Although high, these correlations remained below the 0.96 threshold, supporting the empirical differentiation among the evaluated dimensions. The strong correlation between Fun and Arousal suggests that items assessing enjoyment and excitement are closely related, possibly reflecting a common dimension of emotional pleasure.

Reliability analyses indicated adequate levels across the four subscales. Cronbach’s alpha coefficients were 0.86 for Fun, 0.83 for Arousal, 0.77 for Curiosity, and 0.90 for Acceptance. McDonald’s omega coefficients yielded equivalent values (*ω* = 0.87, 0.83, 0.79, and 0.91, respectively), indicating solid internal consistency across all dimensions.

Regarding the Average Variance Extracted (AVE), all factors met or approached the recommended threshold of 0.50, with values ranging from 0.47 (Curiosity) to 0.58 (Acceptance), providing further support for the convergent validity of the model.

## Second study

The sample comprised 1,000 young people aged between 18 and 29 years (*M* = 23.5, SD = 3.47). It was derived from a larger dataset collected by the research company 40 dB DATA, S.L. Participants were recruited using a stratified sampling procedure, with strata defined by sex, age group, educational level, and social class. Of the total, 50.1% (*n* = 501) were women and 49.9% (*n* = 499) were men. Regarding education and current status, 29.7% had completed secondary education, 31.2% high school, and 30.8% university studies. In terms of social class, 57.1% identified as lower-middle and 26.7% as upper-middle. All participants were Spanish nationals.

The *Attitudes toward Pornography Scale* (EAP) (Monferrer and Flor, 2015) was administered using a 6-point Likert scale (1 = Strongly Disagree, 2 = Disagree, 3 = Somewhat Disagree, 4 = Somewhat Agree, 5 = Agree, 6 = Strongly Agree). Higher scores indicate more favorable attitudes toward pornography. In this study, the intermediate category of the scale was removed. As Kulas and Stachowski (2009) note, the midpoint in odd-numbered scales often lacks a clear meaning and may serve as a ‘dumping ground’ for difficult responses. Similarly, Bisquerra and Pérez-Escoda (2015) warn that the neutral option may be unconvincing when participants do not find a response aligned with their perception. To avoid conflating ambivalence with semantic confusion, a forced-choice format was adopted, encouraging more defined stances and enhancing the interpretative validity of the data.

The additional items assessing pornography consumption and interest in specific types of sexual content were developed *ad hoc* for this study, based on previous literature on pornography typologies and content analyses distinguishing between mainstream and more transgressive forms of pornography. These included: (1) *I have consumed pornography or online sexual content (texts, images, videos, or downloads of sexual images available on websites and platforms)*; *If I were to consume pornography …:* (2) *I would seek content depicting explicit sexual acts with penetration*; (3) *I would want to view sexual content involving bondage, domination, submission, or sadomasochism*; (4) *I would be attracted to content involving violent or non-consensual sexual practices*; and (5) *I would be interested in fetishistic content* (e.g.*, specific body parts, feet, objects, latex, etc.*). The use of different verbs (e.g., “*I would seek*,” “*I would want to view*,” *“I would be interested in*”) was intentional, aiming to capture varying degrees of subjective interest and behavioral intention. These items were rated on a 5-point Likert scale (1 = Not at all likely, 2 = Slightly likely, 3 = Likely, 4 = Quite likely, 5 = Very likely).

## Data analysis

A mixed approach was applied, combining descriptive techniques, cluster analysis, and non-parametric tests, as the variables did not follow a normal distribution. Continuous variables were standardized as Z-scores to ensure comparability in the cluster analysis (Hair et al., 2014). Cluster analysis was performed with the k-means algorithm. The optimal three-cluster solution was selected using the Gap statistic (Tibshirani et al., 2001), balancing interpretability and statistical adequacy. Partition quality was evaluated with the silhouette index (mean = 0.27), indicating moderate differentiation (Rousseeuw, 1987). Cluster validity was further supported by Kruskal–Wallis tests, with effect sizes reported as ε^2^ and interpreted according to Cohen’s (1988) benchmarks: 0.01 (small), 0.06 (medium), 0.14 (large).

Gender distribution across clusters was examined using Pearson’s χ^2^ with Cramér’s V as effect size (0.10 small, 0.30 medium, 0.50 large; Cohen, 1988). Finally, Mann–Whitney U tests compared men and women on content types and attitude scales, with effect sizes expressed as rank-biserial correlations (*r*), following Cohen’s (1988) conventions: 0.10 small, 0.30 medium, 0.50 large.

## Results

A total of 28.5% reported never having consumed pornography, while nearly half fell within the range of occasional or moderate use (“rarely” or “sometimes,” 51.2%), and 20.3% indicated frequent use (“quite often” or “frequently”). The Mann–Whitney U test revealed statistically significant differences in pornography consumption between men and women (W = 156.903, *p* < 0.001), with a small-to-moderate effect size (r = 0.230). The findings indicate that men report higher levels of pornography consumption compared to women. Women were overrepresented in the non-consumption category (37.1% versus 19.8% among men), whereas men predominated in the higher-frequency categories: 16.6% reported consuming “quite often” and 9% “frequently,” compared to 11.4 and 3.6% of women, respectively. Moderate or occasional consumption, encompassing the categories “rarely” and “sometimes,” emerged as the most prevalent pattern in the general population. Approximately half of the respondents fell within this range, with 54.5% of men and 47.9% of women classified accordingly. Although moderate use was the dominant pattern for both sexes, notable differences emerged in the internal distribution: men were more likely to report intermediate frequency (“sometimes,” 32.7%), whereas women more frequently reported highly sporadic use (“rarely,” 25.9%).

The descriptive analysis of the intention or preference to search for different types of content revealed that, for the total sample, explicit sexual acts involving penetration showed the highest mean level of interest (*M* = 2.58, SD = 1.26), emerging as the most preferred content among the items evaluated. The next most frequently preferred category was BDSM (bondage, domination, submission, or sadomasochism) (*M* = 2.39, SD = 1.32). In contrast, the lowest mean intention to search was observed for violent or non-consensual practices (*M* = 2.13, SD = 1.29) (Table 2).

**Table 2:** Gender differences in pornography contents and attitude scales.

Item	Total	Men	Women	*r*
	M (SD)	M (SD)	M (SD)	
Explicit content and penetration	2.58 (1.26)	2.78 (1.26)	2.37 (1.24)	0.161
BDSM	2.39 (1.32)	2.58 (1.30)	2.20 (1.32)	0.141
Violent or non-consensual practices	2.13 (1.29)	2.39 (1.33)	1.87 (1.20)	0.201
Fetishism	2.18 (1.31)	2.47 (1.33)	1.89 (1.22)	0.220
Attitudes scale	3.41 (0.76)	3.60 (0.65)	3.22 (0.81)	0.253^**^
Fun subscale	3.46 (0.87)	3.66 (0.78)	3.26 (0.91)	0.220^**^
Arousal subscale	3.51 (0.90)	3.71 (0.79)	3.31 (0.95)	0.212^**^
Curiosity subscale	3.32 (0.10)	3.56 (0.89)	3.08 (1.04)	0.217^**^
Acceptance subscale	3.35 (0.93)	3.47 (0.89)	3.22 (0.96)	0.125^**^

M, mean; SD, standard deviation; *r*, effect size from Mann–Whitney *U*-test. *p < 0.01.

En cuanto a las diferencias por sexo, se identificaron consistentemente diferencias significativas en la With respect to sex differences, statistically significant differences were consistently identified across all types of content evaluated (*p* < 0.001 in all cases), with men showing a greater likelihood of interest or preference for all categories compared to women. The magnitude of these differences, assessed through point-biserial correlation (*r*), was classified as small for explicit penetration content (*r* = 0.16) and BDSM (*r* = 0.14). In contrast, gender differences reached a small-to-moderate effect size for violent or non-consensual practices (*r* = 0.20) and fetishism (*r* = 0.22).

On the global scale of attitudes toward pornography, the total sample obtained a mean score of *M* = 3.41, SD = 0.76 (range 1–6), indicating overall neutral to slightly favorable positions. Men reported significantly higher scores (*M* = 3.60, SD = 0.65) than women (*M* = 3.22, SD = 0.81), *U* = 159,966, *p* < 0.001, with a small-to-moderate effect size (*r* = 0.253).

Regarding the subscales, the highest mean scores were observed in *Arousal* (*M* = 3.51, SD = 0.90) and *Fun* (*M* = 3.46, SD = 0.87), indicating these two dimensions were the most prevalent attitudes. The *Acceptance* subscale registered a mean of *M* = 3.35 (SD = 0.93), which positioned the overall perception of the sample as neutral or ambivalent. In terms of gender differences, men consistently scored higher than women across all four dimensions. In *Fun*, *Arousal*, and *Curiosity*, these differences were statistically significant and reflected a small-to-moderate effect size (*r* ≈ 0.212–0.220). Finally, the gender gap for *Acceptance* (*M* = 3.35, SD = 0.93) was also significant (U = 142,990, *p* < 0.001), but corresponded to the smallest effect size of all subscales (*r* = 0.125), indicating that this dimension shows the most similar perceptions between men and women.

**Figure 1:**
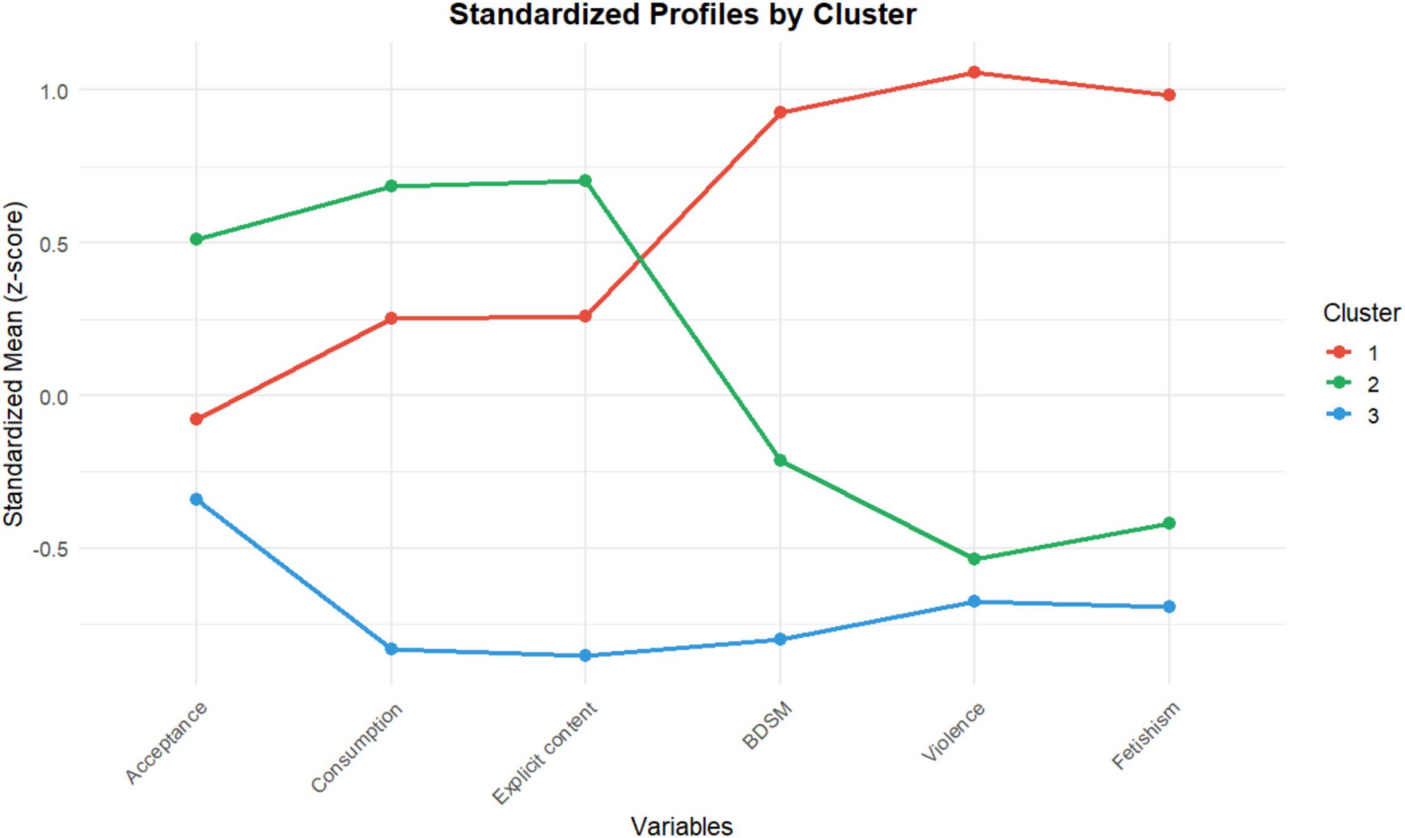
Cluster profile plot.

Cluster 1 was characterized by the highest intention within the sample to seek non-conventional content such as *violent or non-consensual practices* (Z = 1.06; *M* = 3.50), *fetishism* (Z = +0.98; *M* = 3.47), and *BDSM* (Z = +0.93; *M* = 3.62). In parallel, this cluster reported occasional to low-moderate levels of self-reported consumption (*M* = 2.77, SD = 1.20), a frequency slightly above the overall sample mean. With regard to social *acceptance* (*M* = 3.28, SD = 0.69), attitudes were neutral or ambivalent, closely aligned with the global mean of the sample (*M* = 3.28; Z ≈ −0.08) (Table 3 and Figure 1).

**Table 3:** Differences across clusters.

Variable	Cluster 1	Cluster 2	Cluster 3	ε^2^
	(*n* = 348)	(*n* = 286)	(*n* = 348)	
	M (SD)	M (SD)	M (SD)	
Acceptance subscale	3.28 (0.690)	3.83 (0.909)	3.03 (1.017)	0.123^*^
Pornography consumption	2.77 (1.203)	3.29 (0.868)	1.45 (0.649)	0.431^*^
Explicit penetration	2.91 (1.167)	3.47 (0.893)	1.50 (0.761)	0.447^*^
BDSM	3.62 (0.957)	2.10 (1.027)	1.33 (0.664)	0.567^*^
Violent or non-consensual content	3.50 (0.984)	1.44 (0.628)	1.26 (0.576)	0.656^*^
Fetishism	3.47 (1.056)	1.63 (0.830)	1.27 (0.605)	0.579^*^

M = mean; SD = standard deviation; ε^2^ = effect size from Kruskal–Wallis’s test. *p < 0.01.

Cluster 2 presented the highest scores in self-reported consumption frequency (Z = 0.68, *M* = 3.29) and in the attitude of social acceptance (Z = 0.51, *M* = 3.83). Their search intention was primarily focused on explicit penetration content (Z = 0.70, *M* = 3.47), where they also scored significantly above the overall sample mean. In contrast, this cluster displayed a clear rejection or low intention toward non-conventional content: their interest in BDSM, fetishism, and particularly in violent or non-consensual practices was consistently below the sample average.

Cluster 3 represented the group with the lowest scores across all variables evaluated (Z ranging from −0.83 to −0.34). Its defining feature was a markedly low frequency of consumption (Z ≈ −0.83, *M* = 1.45, on a scale where 1 = “Never”), suggesting that most members identified as either non-consumers or very sporadic consumers. Consistent with this low level of consumption, the cluster showed the lowest intention to search for specific content, with raw means approaching the minimum of the scale (M ≈ 1.27–1.50). Finally, this cluster reported the lowest score in social acceptance (Z ≈ −0.34, *M* = 3.03), highlighting a general perception of pornography as socially less acceptable and reinforcing their low level of involvement.

Sex distribution differed significantly across clusters (Cramer’s V = 0.255, *p* < 0.001), indicating a small-to-moderate association. Regarding group composition, men were overrepresented in Cluster 1 (59.8%) and Cluster 2 (58.4%), whereas women constituted the majority in Cluster 3 (67.5%).

The Kruskal–Wallis’s test revealed significant differences across clusters in all variables analyzed (*p* < 0.001), with moderate effect sizes for acceptance (ε^2^ = 0.12) and large to very large effect sizes for consumption and content types (ε^2^ = 0.43–0.66). These findings confirm that the clusters are not merely statistical artifacts but instead reflect clearly differentiated profiles, particularly in relation to the types of pornography that participants found most appealing (Table 3).

## Discussion

The results of the first study showed that the four-factor model demonstrated good and parsimonious fit, with significant loadings confirming convergent validity and explaining between 21 and 82% of item variance. Factor correlations were high yet distinct, with Fun and Arousal showing the closest association. Reliability indices ranged from good to excellent, supporting the internal consistency and utility of the EAP as a valid instrument.

Regarding the second study, findings indicate that pornography use is widespread among young people in Spain, although notable differences emerge across frequency categories. Seventy-one point 5 % of the sample reported pornography use, a prevalence consistent with previous studies (Arredondo Quijada et al., 2025; Ballester et al., 2022). However, higher prevalence rates have been reported in other research, reaching up to 93% in specific samples (Canet Benavent and Martínez Martínez, 2023; Durante, 2022; Martínez-Fernández et al., 2025). In terms of frequency, the results corroborate the literature in highlighting a marked gender divide: men predominate in the higher-frequency categories, whereas women are more often represented among non-users or sporadic users (Ballester et al., 2022; Díaz Hernández et al. 2024; García and Pollán, 2024; Gutiérrez García, 2024; Instituto de la Juventud (INJUVE), 2021). Although 47.9% of women’s consumption falls within occasional categories (“rarely” and “sometimes”), and despite evidence of a substantial increase in women’s participation in recent years (Martinez-Serra and Cardenal, 2025), sporadic use continues to outweigh habitual use in this group, while men remain predominant in both frequency and intensity of consumption (Ballester et al., 2022; Díaz Hernández et al., 2023; FAD Juventud, 2023; Gutiérrez García, 2024; Sanz-Barbero et al., 2023). Although women were overrepresented among non-users and sporadic users, the presence of a subgroup of women reporting relatively frequent consumption suggests that female pornography use is not homogeneous and may respond to diverse motivations and contexts, warranting further qualitative and quantitative exploration.

The analysis of content preferences showed that explicit sexual acts involving penetration were the most highly valued type, followed by BDSM, whereas violent or non-consensual content elicited the lowest intention to search. In the present study, the distinction between “conventional” and “transgressive” content is based exclusively on the degree of prevalence and normalization of different practices within mainstream pornographic platforms, and does not imply moral, clinical, or attitudinal evaluations of those practices. Across all categories, significant sex differences were observed, with men showing a greater likelihood of interest than women. The magnitude of these differences was small for conventional content (penetration, BDSM) and small-to-moderate for violent practices and fetishism, indicating a greater tolerance among men toward more transgressive content. These findings are consistent with previous studies reporting higher male consumption of violent or incestuous pornography (Colom et al., 2024; Merlyn et al., 2020), the presence of a relevant segment of young people accessing violent pornography (FAD Juventud, 2023), and more negative perceptions of paraphilic pornography among women (Prantner et al., 2024). Taken together, the results support the hypothesis that men show greater willingness to consume violent or non-conventional content.

Similarly, the results of this study show that men scored higher than women both on the subscales and on the overall scale of attitudes toward pornography, with small-to-moderate effect sizes. The gender difference was smallest for the *Acceptance* subscale; however, consistent with the proposed hypothesis, men also reported higher levels of acceptance in this domain, albeit with the weakest effect size. Beyond gender differences, positive attitudes toward arousal and fun in the total sample align with previous research highlighting masturbation, satisfaction, and sexual exploration as the main motives for pornography consumption among Spanish youth (Ballester et al., 2022; FAD Juventud, 2023; Martínez-Fernández et al., 2025). Regarding self-perceived evaluations of pornography’s effects, available evidence indicates that young people themselves emphasize positive personal outcomes, identifying pleasurable masturbation, curiosity satisfaction, and sexual learning as primary benefits. Moreover, pornography is often regarded as a normal part of their sexual development due to its accessibility and perceived utility (Ballester et al., 2022; Peterson et al., 2023; Prantner et al., 2024). Nonetheless, when evaluations extend to social or ethical dimensions, more critical and ambivalent attitudes emerge. Negative perceptions center on violence and the denigration of women, framing pornography as an industry that fosters sexual exploitation, sexism, abuse, and dominance, while simultaneously normalizing rape culture (Colom et al., 2024; FAD Juventud, 2023; Idoiaga-Mondragon et al., 2025; Peterson et al., 2023). Content is perceived as male-centered, with strong criticism directed toward aspects considered degrading or disrespectful toward women (Idoiaga-Mondragon et al., 2025; Fernández-Ruiz et al., 2023). Young people also question the unrealistic nature of pornographic content and so-called “pornographic bodies,” noting a marked contrast between what is depicted and real-life experiences, which generates false expectations about sex (Idoiaga-Mondragon et al., 2025; Noll et al., 2022). Consistently, studies confirm that most young people recognize inequality and violence in pornography (Arredondo Quijada et al., 2025; FAD Juventud, 2023). While they acknowledge certain personal benefits, they also report that such content can provoke guilt and discomfort when it conflicts with personal or cultural values (Noll et al., 2022; Peterson et al., 2023). Men’s perceptions are significantly lower across all these critical aspects, underscoring women’s greater sensitivity and critical stance. However, ambivalence does not always translate into outright rejection: endorsement of rape myths was higher even among those who expressed neutral rather than explicitly negative attitudes, suggesting a problematic form of neutrality (Noll et al., 2022).

The cluster analysis complemented these findings by clearly distinguishing young non-consumers of pornography from those who do consume it. Furthermore, within the group of consumers, two differentiated profiles emerged, indicating that youth consumption is not a homogeneous phenomenon but rather one that is organized into subgroups with specific patterns (Maitland and Neilson, 2023; Prantner et al., 2024).

The non-consumer group (Cluster 3) is characterized by low or no pornography use, low levels of social acceptance, and little interest in any type of sexual content, and it is clearly overrepresented by women (67.5%). This profile reflects disengagement or indifference toward pornography—a critical or abstinent group—consistent with the literature documenting lower consumption among women and more negative perceptions of pornography (Ballester et al., 2022; Colom et al., 2024; FAD Juventud, 2023; Fernández-Ruiz et al., 2023; Peterson et al., 2023).

With respect to consumption profiles, men were overrepresented, consistent with prior evidence of higher use among men than women. Yet, within this broader pattern, distinct profiles emerged, underscoring the heterogeneity of consumption behaviors. Cluster 2 represents the profile of *Frequent* and *Conventional* Consumers, defined by the highest frequency of use within the sample and a clear preference for explicit sexual content, with little interest in non-conventional material. This pattern is accompanied by the highest scores on the *Acceptance* subscale, positioning this group as the most engaged and socially affirmative with respect to pornography. The literature supports this association, showing that positive attitudes toward pornography correlate with greater use (Noll et al., 2022). Members of this cluster also reported high levels of social acceptance of consumption, directed primarily toward explicit and conventional content, consistent with the perception of personal benefits related to pleasure, exploration, and sexual education (Bhuptani et al., 2024; Ballester et al., 2022; Peterson et al., 2023; Prantner et al., 2024). These findings reflect the cultural normalization of pornography, perceived as a legitimate and socially integrated practice in youth sexual development, and as a tool for pleasure, information, and learning—particularly in contexts marked by deficiencies in sexual education (Arredondo Quijada et al., 2025; Peterson et al., 2023; Bhuptani et al., 2024).

On the other hand, cluster 1 groups together young people whose pornography consumption exceeds the sample mean and who display a clear orientation toward transgressive or non-conventional content such as BDSM, violence, and fetishism. This pattern reflects a profile of explorers of alternative sexual practices, confirming the existence of a subgroup with greater tolerance or preference for material that departs from the mainstream and is associated with social risks. The literature supports this characterization, as several studies document that a proportion of men consume violent pornography and show greater tolerance for extreme material (Ballester et al., 2022; Colom et al., 2024; Díaz Hernández et al., 2024; Merlyn et al., 2020), while a considerable number of young people are at least occasionally exposed to violent sexually explicit content (FAD Juventud, 2023). Likewise, attraction to BDSM and fetishism has been linked to the association of paraphilic pornography with representations of dominance, submission, and sexual violence, particularly among women (Prantner et al., 2024). Regarding attitudes, members of this cluster exhibited an ambivalent pattern, close to the overall sample mean. The absence of a clear critical stance in this group may be associated with different interpretative processes, including normalization, indifference, or entertainment-oriented engagement with pornographic content (Colom et al., 2024; Noll et al., 2022).; however, the present data do not allow disentangling these mechanisms or inferring attitudes toward sexual violence or rape myths. Importantly, interest in or consumption of BDSM-related content should not be conflated with acceptance of sexual violence or rape myths, as consensual BDSM practices are conceptually distinct from coercion or abuse, and such distinctions were not assessed in the present study. Previous literature has described patterns of indifference or normalization in relation to repeated exposure to transgressive pornographic content; however, the present findings do not allow direct inferences about these mechanisms, which lie beyond the scope of the current data.

At the same time, this profile raises gender-related risks. Men’s exposure to pornography depicting bondage or aggression has been correlated with more negative attitudes toward women. Moreover, such content has been associated with elevated levels of hostile sexism in both sexes (Sanz-Barbero et al., 2023) and with the promotion of objectifying views of women (Mestre-Bach et al., 2024). In addition, this type of material emphasizes male gratification and the commodification of women, thereby normalizing dynamics of inequality (Idoiaga-Mondragon et al., 2025). Regular consumption has also been linked to a greater risk of engaging in sexually violent practices (Léon et al., 2025). Complementary evidence shows that higher frequency of pornography consumption is positively associated with the dehumanization of women, as men with elevated levels of use tend to perceive women merely as sexual objects (Zhou et al., 2021). According to previous research, these patterns have been interpreted in light of social learning theory (Zarei et al., 2025), which explains how repeated observation of behaviors may be internalized and subsequently imitated by individuals. In the case of pornography, continued exposure to explicit content—particularly of a violent or transgressive nature—facilitates the incorporation of sexual scripts that normalize male dominance and female objectification. According to previous research, social learning frameworks have been used to interpret associations between repeated exposure to certain pornographic contents and more neutral or ambivalent attitudes toward sexual violence; nevertheless, such interpretations cannot be derived from the present study and should be considered cautiously.

Taken together, the results supported all hypotheses: men reported higher frequency of use, more favorable attitudes toward pornography, and greater willingness to consume transgressive content, while the cluster analysis confirmed the heterogeneity of usage patterns, highlighting differentiated profiles of non-consumers, conventional consumers, and transgressive consumers.

These findings underscore the need to consider not only the frequency but also the type of consumption in the design of preventive interventions. Continued exposure to transgressive pornography not only shapes individual attitudes but also reinforces structural dynamics of gender inequality. Further research is required to deepen understanding of the relationship between pornography use, sexist attitudes, and constructions of masculinities, in order to clarify the mechanisms through which such consumption may foster acceptance of sexual violence and the perpetuation of traditional gender roles.

Complementarily, the results confirm that pornography constitutes a phenomenon of major social relevance for Spanish youth, characterized by high prevalence, marked gender differences, and notable heterogeneity in usage profiles. While young people tend to emphasize personal benefits such as pleasure, curiosity, or sexual learning, they also express critical attitudes toward violent, sexist, and objectifying content, reflecting the ambivalence highlighted in the literature. The identification of differentiated profiles highlights that consumption is not homogeneous but rather responds to diverse motivations ranging from cultural normalization to tolerance of transgressive content with social and gender implications. Within this context, the Pornography Attitudes Scale (EAP) emerges as a robust tool to capture both the positive and critical dimensions of pornography.

## Limitations and future research

This study presents several limitations that should be acknowledged. First, the cross-sectional design prevents the establishment of causal relationships between the variables analyzed; therefore, the results must be interpreted in terms of association. Second, the use of self-reports may be influenced by social desirability bias, particularly in relation to a sensitive issue such as pornography. In addition, although the sample was stratified by sex, age, educational level, and social class, it consisted exclusively of young people of Spanish nationality, which limits the generalizability of the findings to other age groups and cultural contexts.

With regard to future research, it would be relevant to employ longitudinal designs to examine the evolution of consumption patterns and their effects over time. Likewise, it is necessary to complement quantitative data with qualitative methodologies that explore the subjective experience and meanings attributed to pornography consumption. Finally, it would be pertinent to investigate the interaction between pornography use, sexist attitudes, and constructions of masculinities, with particular attention to the role of exposure to violent content in the acceptance of rape myths and the perpetuation of traditional gender roles.

## Data Availability

The raw data supporting the conclusions of this article will be made available by the authors, without undue reservation.

## Footnotes

1
https://www.pornhub.com/insights/yir-2021#Age-Demographics
